# Life-history strategy, adverse environment, and justification of life-ending decisions

**DOI:** 10.3389/fpsyg.2025.1568204

**Published:** 2025-07-21

**Authors:** Shaolingyun Guo, Hui Jing Lu

**Affiliations:** Hong Kong Polytechnic University, Kowloon, Hong Kong, Hong Kong SAR, China

**Keywords:** life history, adverse environment, life-ending decisions, cognitive judgment, world values survey

## Abstract

**Objective:**

Evidence remains limited regarding the interplay between childhood environment, as reflected by life-history calibration, and the current environment, as well as their combined influence on cognitive judgments about life-ending decisions. Drawing on life-history theory, the present study aims to (1) examine whether life-history trade-offs along the fast-slow continuum are associated with the subjective justification of suicide and assisted suicide (euthanasia practices), and (2) explore whether the current environment moderates this relationship.

**Methods:**

In Study 1, a vignette-based questionnaire was administered to Chinese young adults (*N* = 147) to examine the relationships among life-history traits, current environmental adversity, and the subjective justification of life-ending behaviors. In Study 2, these hypotheses were further tested using cross-national data from the World Values Survey (*N* = 6,766). Structural equation modeling was employed in both studies to analyze the proposed associations.

**Results:**

Findings from Study 2 indicated that individuals who adopted a slow life-history strategy were less likely to subjectively justify life-ending behaviors. Furthermore, results from both studies demonstrated that the relationship between life-history strategy and the justification of life-ending decisions was moderated by current environmental adversity.

**Conclusion:**

These findings underscore the influence of life-history orientation on cognitive judgments related to life-ending decisions and highlight the moderating role of current environmental conditions. Implications for future suicide intervention programs are discussed.

## Introduction

Humans have the instinct to survive (Miller, 2019). However, when individuals face extrinsic environmental threats (e.g., epidemic disease, natural disasters, and harsh childhood living environments; Brumbach et al., 2009) or unpredictable environments (e.g., uncertain fluctuations in threats and childhood unpredictability characterized by frequent changes or inconsistency in the presence, relationships, and behavior of caretakers and family; Belsky et al., 2012), the psychological mechanisms that influence and regulate human development and behavior may vary (Stearns, 1992). In the past few years, emerging environmental and ecological concerns have gained increasing attention in dealing with shrinking resource and sustainability, deforestation, global warming, harmful chemical pollution, etc. (Haque, 2000). This creates survival pressures that can hinder effective survival-oriented behavior, and potentially survival itself, unless they are effectively managed (Pyszczynski et al., 2015). For example, the outbreak of the Coronavirus and its rising mortality rate have generated widespread concern and anxiety due to the stark exposure to death, accompanied by other unsettling reactions at both personal and societal levels. Ultimately, everyone will confront life-ending issues at some point, and throughout their lives, many people will witness others facing death and the dying process. Because the acquisition of energy and resources is constrained by environmental factors, all living organisms face the fundamental challenge of allocating limited time, energy, and resources among the various tasks necessary for survival and reproduction (Ellis et al., 2009; Griskevicius et al., 2011b). This study applies life-history (LH) theory to human psychology to examine how environment-driven LH trade-offs and their psychological manifestations influence judgments about life-ending decisions. A cross-sectional sample (Study 1) and cross-cultural secondary data from eight countries (Study 2) were used to investigate the influence of LH-related manifestations on attitudes toward life-ending decisions, including suicide and assisted suicide (euthanasia), as well as the moderating role of current environmental contingencies in shaping individuals’ LH-related profiles.

Life-ending decisions refer to choices to hasten death by active or passive means, initiated by or taken on behalf of an individual (Harris, 2003). Suicidal behaviors or suicide attempts, as significant forms of life-ending behavior, have received increasing attention in both research and public awareness. Previous cohort studies have provided evidence that suicide and suicide attempts are strongly associated with the genetic heritability of psychiatric disorders (Brent, 1996), changes in gene expression in response to environmental cues (Turecki et al., 2019), and early life adversity (Brezo et al., 2009). The risk factors for suicide result from the interaction of biological, clinical, psychological, social, cultural, and environmental influences (Turecki et al., 2019). It is also crucial to further understand suicide awareness, the justification of suicidal behaviors, and the distal or mediating factors that may increase the risk of suicidal ideation or behavior. Assisted suicide, which may have a life-shortening effect, is part of contemporary euthanasia and end-of-life care (Rietjens et al., 2012). Because it is not possible to assess suicidal behavior psychologically in individuals who have died by suicide, few theoretical models have been developed to fully understand life-ending behaviors (Prinstein, 2008). Beyond biological factors, previous studies have identified a range of risk factors for suicide, suicidal ideation, and attempts, including impulsivity (Brent et al., 1994), family conflict (Bastia and Kar, 2009), social exclusion and isolation (Trout, 1980), social withdrawal, living alone, and limited social support (Turvey et al., 2002). The goal of this study is to build upon the existing empirical foundation by delineating evolutionary connections among risk factors and the life-ending behavior. We propose that decisions regarding life-ending behaviors are associated with fast versus slow LH strategies and are influenced by adverse environmental conditions.

### Life history (LH) theory

Life history (LH) theory addresses the trade-offs involved in allocating time and resources across an organism’s lifespan to various functions, as well as the influence of the local environment on achieving an optimal allocation balance (Kaplan and Gangestad, 2005). Individuals are often adaptive, adjusting their LH allocations in response to cues about environmental conditions and their own state (Frankenhuis, 2019). In humans, critical ecological cues for calibrating LH strategy include both intrinsic and extrinsic mortality risks (Chang et al., 2019). Extrinsic mortality refers to the risk of death from external factors, such as aging, that is equally shared by all members of a population (Stearns, 1992), and does not account for mating or parenting effort (Quinlan, 2010). On the other hand, intrinsic morality is the probability of death resulting from an individual’s allocation of resources, such as those devoted to somatic maintenance and reproductive effort (Quinlan, 2010). As perceived mortality risk increases, individuals tend to reduce energy investment in long-term health as an adaptive response to their environment (Pepper and Nettle, 2014). LH trade-offs are based on the idea that individuals differ in how they allocate bioenergetic and material resources between somatic effort (resources devoted to continued survival) and reproductive effort (resources devoted to mating and parenting) (Jonason et al., 2016). The development of LH theory addresses the ecological challenges posed by the environment while accounting for the intrinsic constraints of the organism (Stearns, 2000). Thus, patterns of resource allocation can be described as LH strategies, which help explain behavioral and psychological differences between individuals in specific environments (Barbaro and Shackelford, 2016).

Although researchers initially used LH theory to explain species-level differences, it has also proven useful for understanding variation within species (André and Rousset, 2020). Studies have shown that humans, like other species, follow developmental patterns shaped by trade-offs along a fast–slow continuum (e.g., Belsky et al., 2012; Quinlan and Quinlan, 2007). Fast LH strategists prioritize current reproduction over future reproduction, investing less in somatic maintenance, whereas slow LH strategists devote more time and energy to growth and maintaining health (Belsky et al., 2012; Ellis et al., 2009). When applied to psychology, LH theory encompasses not only classical traits such as the timing of maturation and reproduction, but also psychological variables including risk attitudes, the ability to delay gratification, prosociality, religiosity, optimism, and others (Buss, 2009; Figueredo et al., 2005). The continuum from short-term (fast) LH strategies, which emphasize present-focused behavior, to long-term (slow) LH strategies, which emphasize future-focused behavior, is influenced by environmental cues signaling harshness and/or unpredictability, affecting the covariation of many human LH traits (Belsky et al., 2012; Griskevicius et al., 2011a). A growing body of experimental work demonstrates that local environmental conditions are correlated with how individuals navigate LH trade-offs (e.g., Belsky et al., 2012; Chisholm, 1993; Nettle, 2010). More generally, adaptive LH trade-offs require the integration of multiple traits and often exhibit coordinated plasticity in response to environmental conditions (Braendle et al., 2011; Roff, 2002). For example, ‘faster’ LH strategies often result from exposure to harsh or unpredictable environments, either individually or collectively (Ellis et al., 2009). Not only can individual LH traits be plastic, but the correlations between traits can also change, with different environments altering the slope and/or direction of these relationships (Stearns, 1992). Previous research has indicated that environments characterized by high morbidity and mortality influence LH trade-offs, as extrinsic risks orient LH behavioral manifestations toward immediate survival goals (Chang et al., 2019; Gordon, 2021). In response to local socio-ecological conditions, LH trade-offs along the fast–slow continuum depend on how individuals acquire energy and optimize resource expenditures in the face of environmental risks.

### LH approach to life-ending risk factors

The association between fast–slow LH strategies and risk factors for life-ending behaviors is especially pertinent to the study of attitudes toward such behaviors. Previous LH literature confirms that LH strategy is shaped by environmental signals experienced during development (Chisholm, 1993), and that LH trajectory is also associated with individual differences in personality (Copping et al., 2013), family relations (Međedović, 2019), social behaviors and family ties (Gladden et al., 2009; Hackman and Hruschka, 2013), and social support (Ziker and Snopkowski, 2020). Researchers have identified clusters of behavioral and psychological correlates associated with slow versus fast LH trajectories in humans (e.g., Bereczkei and Csanaky, 2001; Hackman and Hruschka, 2013). Empirical data confirm that faster LH strategies are more likely to be adopted in perilous, threatening, and resource-limited ecologies (Simpson et al., 2012), whereas slower LH strategies are more common in stable, less threatening, and resource-rich environments (Simpson et al., 2012). A central prediction of these variations is that ecological differences in resources and mortality risks shape the key LH trade-offs, leading to individual differences in fast-slow LH propensities. For example, individuals in threatening ecologies and resource-limited environments tend to exhibit greater risk-taking propensity (Figueredo et al., 2018), impulsivity (Copping et al., 2013), and present-oriented time preference (Gladden et al., 2009). Some empirical data support the hypothesis that risk factors for life-ending thoughts and behaviors exhibit individual differences along a fast–slow LH trajectory (Ziker and Snopkowski, 2020). Several other warning signs for life-ending behaviors, such as signs of acute risk, lack of future plans, and withdrawal from future commitments, have been linked to impulsive decision-making styles and a tendency to discount delayed rewards, both of which are influenced by a faster LH orientation (Dombrovski et al., 2011; Gvion et al., 2015; Klonsky and May, 2010). Stressful family and social environments promote the development of psychological and behavioral response systems characteristic of a fast LH strategy (Promislow and Harvey, 1990). Previous studies on life-ending behaviors have identified several stressful family and social environmental factors, such as family conflicts, deficits in family functioning, and economic constraints, as being associated with suicidal behaviors (Compton et al., 2005; Nazarzadeh et al., 2013). Through the lens of LHT, previous findings have shown that socially deviant traits supporting exploitative tendencies are associated with a faster LH strategy (e.g., Figueredo et al., 2005; Gladden et al., 2009). According to the interpersonal–psychological theory of suicide (Van Orden et al., 2010), these traits, including dimensions of psychopathy such as antisocial, callous, and impulsive characteristics, are related to both suicidal desire and the capability for suicide (Harrop et al., 2017). It has attracted particular research interest that this motivational and cognitive machinery, characterized as fast LH-related socially deviant traits, are adaptive under certain environmental conditions (Gutiérrez et al., 2022). Previous LHT evidence demonstrates that faster LH strategies are characterized by greater present orientation, higher impulsivity, and increased risk-taking, whereas slower LH strategies are associated with less present orientation, lower impulsivity, and greater risk aversion (Wu et al., 2020). When the future is uncertain or unpredictable, or when mortality rates are high, engaging in self-harming behaviors may, paradoxically, improve fitness by prioritizing immediate outcomes (Ziker and Snopkowski, 2020). In such contexts, seeking immediate outcomes can be adaptive due to the cost-effectiveness of resource allocation, and the reverse is true in more stable environments (Bereczkei and Csanaky, 2001). The fast–slow developmental schedule corresponds to a psychological time orientation focused on either the present or the future; thus, a short-term, present-oriented perspective (Chen and Chang, 2016). Suicidality and deliberate self-killing could be considered time-related outcomes in the life course because these actions bring an end to reproductive potential and fitness (Soper, 2018). A previous study on impaired decision-making in suicide found that disrupted future value signals in the ventromedial prefrontal cortex are associated with suicide attempts (Dombrovski and Hallquist, 2017). Thus, we propose that socially deviant traits linked to a faster LH strategy may be associated with specific decision-making processes involved in life-ending acts, such as suicide and euthanasia.

Among the potential risk factors for suicidal intentions and ideations, negative family relationships are particularly influential. Based on recent findings in LHT, we expect that early negative family environments, parental absence, and a lack of social ties and support will be associated with patterns of faster LH behavioral strategies, which involve less stable and more transient pair bonding (Bereczkei and Csanaky, 2001). These predictions have received are supported by previous evidence showing that early-life family stress predicts later manifestations of faster LH strategies, such as increased risky behaviors (Simpson et al., 2012) and greater delay discounting (Kim et al., 2018). Previous empirical data has confirmed the hypothesis that, under conditions of declining family and social support and increasingly stressful socio-environmental conditions, individuals (especially adolescents) experience greater rates of suicidal ideation (Ziker and Snopkowski, 2020). In addition, previous studies have documented associations between suicide and factors such as familial discord, family-related stress, and perceptions of being a burden on the family (e.g., Gould et al., 1996; Van Orden et al., 2010). Under the influence of persistent social and familial stress, LH trajectories may vary in their environmental adaptations related to the decision to engage in suicide or other life-ending behaviors. The goal of this study is to explore how risk factors across multiple domains interact and how variations in LH strategies influence the desire for life-ending behaviors.

### The impact of current adverse environment

Previous empirical findings have identified environmental risk factors that serve as indicators of underlying causal processes leading to life-ending decisions, including environmental risk factors associated with dysfunctional, disorganized, violent, and abusive family environments (Mościcki, 1997), social-environmental variables associated with fluctuations in the income level (Sareen et al., 2011), and disadvantaged in society (e.g., lower educational and socio-economic groups; Daly and Wilson, 2009). Likewise, a substantial body of developmental literature has documented reliable associations between the aforementioned environmental factors and faster LH strategies, including the development of impulsive and risk-taking personality traits (Figueredo et al., 2005) as well as violent-related behaviors (Joyner and Beaver, 2023). According to LH theory, individuals are generally sensitive to certain environmental cues, continue to respond to their current environments (Pepper and Nettle, 2017), and regulate their behaviors accordingly (Del Giudice and Belsky, 2011). The important environmental signals include harshness, which refers to the rates at which external factors cause disability and death at each age in a population, and unpredictability, which reflects the levels of variation in environmental harshness across time and space (Ellis et al., 2009). A stressful environment can be harsh and/or unpredictable, and each environmental dimension may affect future behavioral patterns (Simpson et al., 2012). Accordingly, both harshness and unpredictability present adults with morbidity-mortality risks that select for fast LH strategies (Ellis et al., 2009). For example, exposure to harsh environments, including perceived disparities in resources and income, as well as high external mortality rates, tends to increase involvement in family violence, either as a perpetrator or a victim (Miller et al., 2001). In an unpredictable environment, individuals can either directly experience temporal or stochastic changes, or be exposed to the behaviors of others that are indicative of environmental unpredictability (Ellis et al., 2009). In particular, the behaviors of family members that signal an unpredictable home life (e.g., unreliable parental care and poor family relationships) are linked to unpredictable future decisions (Brumbach et al., 2009). Adopting an evolutionary perspective can provide a deeper understanding of the myriad forces that influence judgments about future health outcomes and the decision-making process. These LH dispositions may shape time preferences, thereby affecting cognitive judgments and decisions about future eventualities. Hence, examining environmental risk factors and how these signals indirectly influence life-ending decisions through the calibration of LH strategy can serve as a stepping stone toward understanding the underlying causes of such decisions.

### Present study

This study contributes to the LH literature on life-ending behaviors and decisions by integrating LHT into an exploratory analysis using both a cross-sectional study and secondary data. We examined the variables of LH strategy, current environmental adversity, and the justification of life-ending decisions using structural models with data from two sources. The hypotheses were tested by analyzing a cross-sectional survey (Study 1) and the World Values Survey (WVS; Study 2). Utilizing two independent datasets enabled us to externally validate our results and assess the robustness of the proposed associations. Additionally, the large, cross-national WVS dataset provided a representative sample of respondents from various countries and geopolitical regions.

The primary goal of the present research is to examine whether LH indicators predict the judgments regarding life-ending behaviors. We hypothesize that individuals who adopt a slower LH strategy will express less subjective justification for life-ending behaviors (H1). Additionally, we explore whether environmental adversity—specifically, harshness and unpredictability—moderates the association between LH strategy and the justification of life-ending behaviors (H2). We expect that a harsh and/or unpredictable environment may exacerbate LH tradeoffs, thereby amplifying the effects of LH traits on the cognitive justification of life-ending behaviors.

## Study 1

### Methods

#### Sampling and recruitment process

This study recruited 204 participants from the local university. Recruitment occurred in several campus locations, and advertisements were placed on publicly accessible social media platforms from May 2021 to August 2021. The study was conducted online and programmed using the Qualtrics software. Participants recruited on campus were asked about their WeChat accounts or email addresses and sent a link to the survey. Participants were recruited through online advertisements to access the study via a web link. All participants accessed to the same questionnaires and received monetary compensation for spending time completing all tasks. The final sample was 147, with 82 male (55.78%) and 65 female (44.22%) participants. The average age was 20.1 years old (*SD* = 1.83). Most participants (*N* = 123; 83.67%) were single or unmarried. Most participants were current undergraduate or graduate students (*N* = 117; 79.59%), whereas others were employed full-time (*N* = 28; 19.04%).

#### Procedure

A vignette survey was distributed to each participant. Participants read information about the general study goals and procedures, such as data handling, anonymity, and voluntariness, and provided written informed consent. Participants first answered questions regarding the LH strategy in the mini-k scheme (Figueredo et al., 2005). After the first part of the survey, each participant read seven vignettes describing suicide and euthanasia situations. After reading the vignettes, participants answered questions about their decisions in probability scores for a hypothetical person’s life-ending scenarios as described in the vignette. Participants then responded to the current environmental adversity scale and demographic questions. Once respondents completed the questionnaires, they were provided with a debriefing form. The study protocol and ethics approval were reviewed and approved by the Institutional Review Board of the authors’ affiliated university. Informed consent was obtained from all participants, and all procedures were conducted in accordance with the ethical guidelines for human subjects at the authors’ institution.

#### Variables

##### Life history strategy

Figueredo et al. (2005) proposed that various indicators of LH strategy converged on a single multivariate construct, the latent K-factor. LH traits were assessed using 20-item Mini-K scales measuring a fast-slow LH dimension (Figueredo et al., 2005). The psychological constructs that could not be directly measured or observed (e.g., LH traits) were quantified as a latent construct/variable. The items were rated on a five-point Likert scale (1 = *strongly disagree*; 5 = *strongly agree*). Higher values indicated a greater inclination toward slow LH traits and vice versa. The Cronbach’s alpha coefficient was reported as 0.77, suggesting that these results meet the standard for internal consistency reliability.

##### Current adverse environment

The current adverse environment was measured in two dimensions: environmental unpredictability (fluctuations in environmental conditions related to social environment instability; Sung et al., 2016) and environmental harshness (limited economic resources and income harshness; Ellis et al., 2009). Luo et al. (2020) obtained four global items of perceived environmental unpredictability. Participants were asked, “*To what extent do you believe that the environment is becoming more dangerous?*” and “*To what extent do you believe the environment is becoming more unsafe?*” and “*To what extent do you believe the environment is becoming more unpredictable?*” and “*To what extent do you believe the environment is getting more uncertain?*” Responses were rated on a seven-point Likert scale (1 = *strongly disagree*; 7 = *strongly agree*), with higher scores indicating higher perceived levels of environmental unpredictability. The Cronbach’s alpha was 0.94, suggesting that these results met the standard for internal consistency reliability. Current environmental harshness was modeled based on the respondent’s household income level and economic resources measured using seven items. Responses were rated on a seven-point Likert scale (1 = *strongly disagree*; 7 = *strongly agree*). Higher scores indicated lower income levels or harsher economic resources. The Cronbach’s alpha was 0.91, which met the standard for internal consistency reliability.

##### Vignettes

Respondents’ agreement with and subjective justification of life-ending decisions were evaluated using vignettes. The primary forms of the vignettes used in this study were derived from life-ending case study vignettes (Maris et al., 1992), suicide attitude vignette experiences (SAVE) (Stiluon et al., 1984), and euthanasia case study vignettes (Kouwenhoven et al., 2012). The vignettes were modified to clarify whether the decision was related to the participant in hypothetical situations (see Appendix A). This was accomplished by using a fictional person who would face life-ending decisions and asking the participants to imagine themselves as that person. The vignettes targeted two life-ending behaviors, suicide, and voluntary euthanasia, with nine scenarios for each behavior, respectively. We used a six-point scale to measure the respondents’ probability scores of committing life-ending behaviors and a five-point scale to assess the justification scores of these life-ending decisions. Participants were asked, “*Do you think this suicidal behavior is justified?*” and “*Do you think euthanasia is justified?*” Higher values indicated higher subjective justification scores for life-ending decisions. The Cronbach’s alpha was 0.94, satisfying the internal consistency reliability standard.

#### Statistical analysis

We used structural equation modeling (SEM) to examine the structural relationships among the latent constructs (e.g., adverse environment) and individual indicators (e.g., environmental harshness and environmental unpredictability). SEM analyzes the variance and covariance of observed variables that represent latent constructs (Motl et al., 2002). For the latent constructs, we used stand-alone measures and constructs as indicators. If a particular item was identified as a poor measure of the latent construct, it was removed from subsequent model development. To test the moderation effect, we applied the product-indicator approach (Kenny and Judd, 1984), in which the latent interaction term is extracted from the products of the indicators of the factors. The structural model included direct paths from the current environmental status toward the justification of life-ending decisions and slow LH traits toward the justification of life-ending decisions. Furthermore, it included indirect paths from the interaction between current environmental status and slow LH traits to justify life-ending decisions. We used probing interactions for a simple slope of residual-centered latent two-way interactions (Preacher et al., 2006).

A correlation matrix was created to examine the relationships between various speeds of slow LH strategies, the current adverse environment, and the justification of life-ending decisions. Multiple indices were used to assess the model fit while testing both the measurement and structural models, including the chi-square to degrees of freedom ratio or *χ2*/*df*, comparative fit index (*CFI*) (Bentler, 1990), Tucker–Lewis index (*TLI*) (Tucker and Lewis, 1973), root mean square error of approximation (*RMSEA*; Steiger, 1990), and standardized root mean square residual (*SRMR*) (Jöreskog and Soerbom, 1993). All statistical analyses were performed using R version 3.5.1.^1^ The SEM model was fitted using the lavaan R package (Rosseel, 2012). The statistical significance level was set at *p* < 0.05. R codes are available in the Supplementary material.

### Results

**Figure 1:**
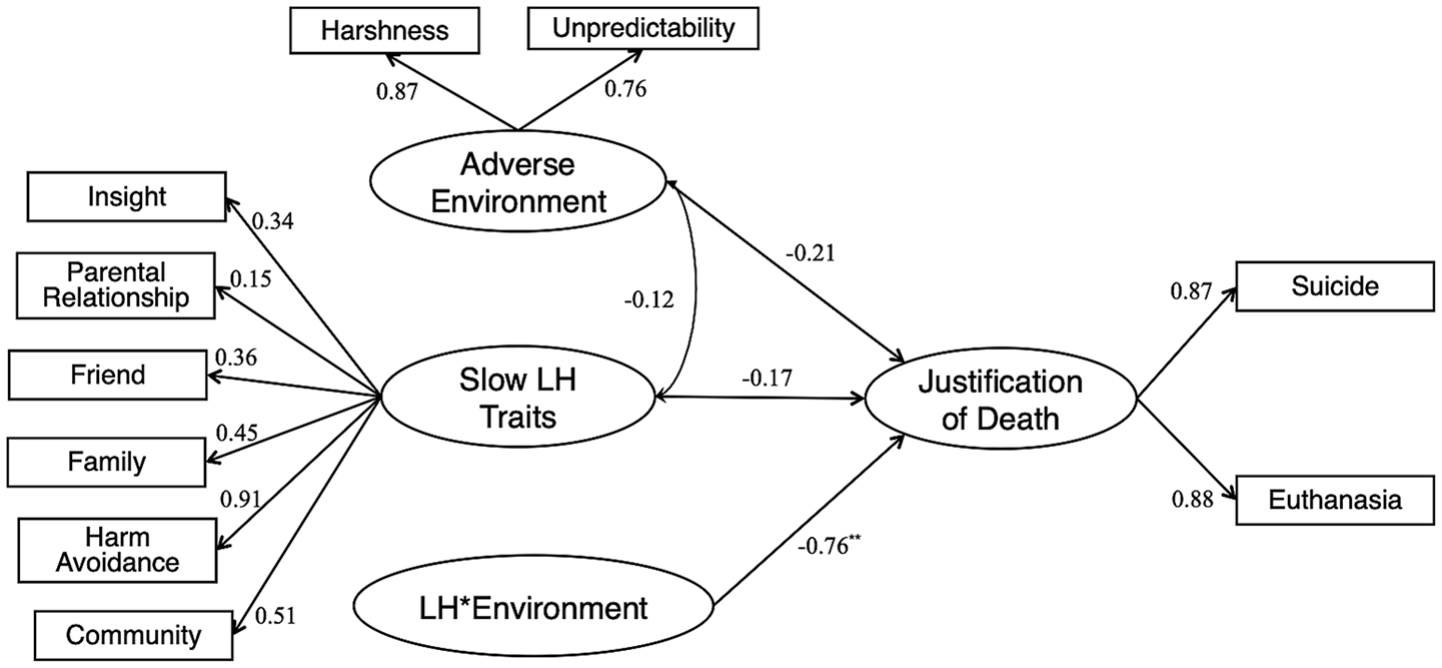
Structural relationships among slow LH traits, current adverse environment and justification of life-ending decisions. ^*^p < 0.05, ^**^p < 0.01, ^***^p < 0.001. (*χ*^2^/*df* = 31.09/28 = 1.11, *CFI* = 0.998, *TLI* = 0.996, *RMSEA* = 0.027, *SRMR* = 0.028). CFI, comparative fit index; RMSEA, root mean square error of approximation; SRMR, standardized root mean square residual; TLI, Tucker–Lewis index. Higher LH values indicate a greater inclination toward slow LH; Higher values for adverse environment indicate increased levels of environmental harshness and unpredictability; Higher justification scores reflect greater subjective justification for life-ending decisions.

**Figure 2:**
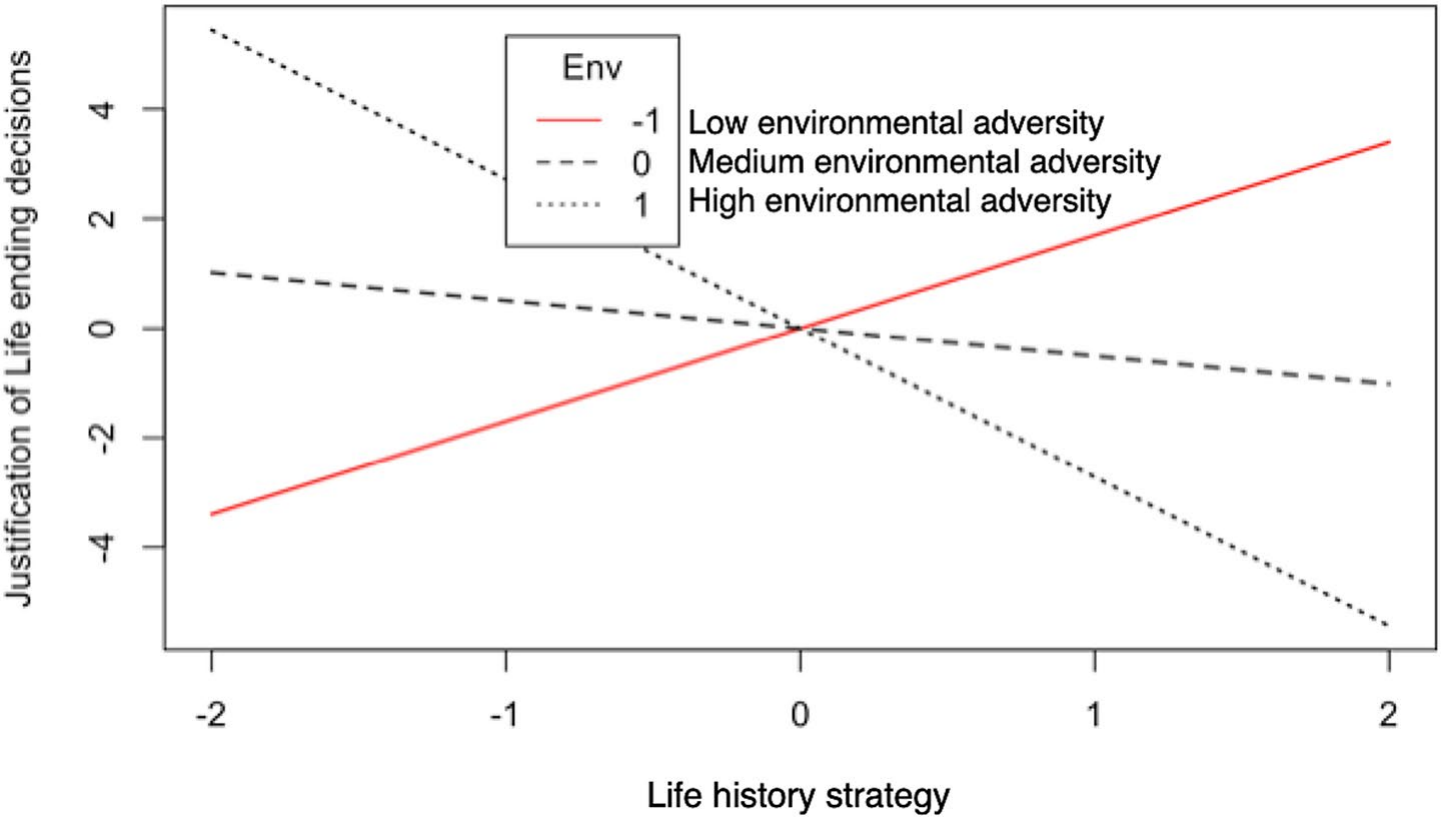
Illustration of the simple-slope analyses for the predicted justification scores of life-ending decisions resulting from the LH-by-current adverse environment interaction (Study 1). Higher LH values indicate a greater inclination toward slow LH; Higher values for adverse environment indicate increased levels of environmental harshness and unpredictability; Higher justification scores reflect greater subjective justification for life-ending decisions.

Descriptive statistics and a correlation matrix for the variables included in the SEM are presented in Table 1. Figure 1 depicts the SEM results. The association between slow LH strategy and the justification of life-ending decisions was non-significant (*β* = −0.17, *p* = 0.579), nor the association between adverse environment and the justification of life-ending behaviors (*β* = −0.21, *p* = 0.344). However, the negative interaction found between LH strategy and adverse environment in this study appeared to reflect the moderation effect resulting from current environmental conditions (*β* = −0.76, *p* < 0.001). As shown in Figure 2, when environmental adversity level was high, individuals who adopt slow LH strategy considered life-ending decisions to be less justifiable; when environmental adversity level was low, individuals who adopt slow LH strategy considered life-ending decisions to be more justifiable. This cross-over interaction indicates that environmental adversity has one kind of effect at the higher level and the opposite kind of effect at the lower level. The SEM had acceptable fit indices, with [*χ^2^* (31.09, *df* = 28) = 1.11, *p* = 0.313], *CFI* = 0.998, *TLI* = 0.996, *RMSEA* = 0.027, *SRMR* = 0.028.

**Table 1:** Correlations among LH, death fear, and the justification of end-of-life decisions.

	Insight	Parent relation	Friend	Family	Harm avoidance	Community	Harshness	Unpredictability	Suicide	Euthanasia
Insight	-									
Parent relation	0.72^***^	-								
Friend	0.78^***^	0.75^***^	-							
Family	0.75^***^	0.69^***^	0.78^***^	-						
Harm avoidance	0.80^***^	0.71^***^	0.83^***^	0.86^***^	-					
Community	0.79^***^	0.69^***^	0.84^***^	0.77^***^	0.88^***^	-				
Harshness	0.77^***^	0.71^***^	0.77^***^	0.77^***^	0.75^***^	0.72^***^	-			
Unpredictability	0.79^***^	0.73^***^	0.79^***^	0.76^***^	0.78^***^	0.79^***^	0.66^***^	-		
Suicide	−0.68^***^	−0.67^***^	−0.66^***^	−0.73^***^	−0.66^***^	−0.69^***^	−0.75^***^	−0.70^***^	-	
Euthanasia	−0.70^***^	−0.69^***^	−0.67^***^	−0.67^***^	−0.69^***^	−0.69^***^	−0.78^***^	−0.71^***^	0.91^***^	-
Mean	4.04	3.93	3.86	3.95	3.79	3.89	3.99	4.28	2.03	1.88
SD	0.82	0.87	0.83	0.84	0.95	0.83	0.68	1.11	0.93	1.05

^*^p < 0.05, ^**^p < 0.01, ^***^p < 0.001.

### Discussion of study 1

Data from the vignette survey confirms that current environmental harshness and unpredictability moderate the association between LH strategy and the justification of life-ending decisions. There is no overall effect of either LH strategy or the current environmental adversity, but a crossover interaction exists (see Figure 2). This cross-over interaction is compatible with the following interpretation. Firstly, it is often adaptive for individuals to adjust their LH strategies based on cues regarding the state of the environment and/or their condition. An adverse environment can be harsh and/or unpredictable, and each of these environmental dimensions may affect the association between LH strategy and cognitive judgments about life-ending behaviors. Further exploration of this interaction effect is needed. Secondly, differential susceptibility theory stipulates that individuals vary in their susceptibility to environmental effects (Belsky, 1997). Because mortality conditions vary widely across environments and time, population variation in LH parameters is mainly traceable to developmental plasticity (Roff, 2002). The plasticity of a specific LH trait may homeostatically buffer the organism against environmentally induced changes so that individuals can adapt to these environmental changes (Stearns, 1992). Suppose the current environment does not match one’s childhood environment regarding harshness and unpredictability. In that case, the inconsistency of one’s past and current environmental uncertainty may hinder the manifestation of one’s previous LH strategy (Zhu et al., 2020). Our finding is consistent with this prediction. When the environment is stable and matches the past environment, slow LH strategists find life-ending decisions less justifiable. In contrast, when the environment is less stable and does not match the past environment, slow LH strategists find life-ending decisions more justifiable. Although testing the impact of long-term calibrations of LH strategy requires proper causal and longitudinal designs, Study 1 reveals the impacts of the current adverse environment on an individual’s LH strategy, which further exhibits some differences in the decision-making of particular life-ending issues.

## Study 2

### Method

#### Data description and sample

The sample was extracted from the sixth wave of the World Values Survey (WVS). Some country-level data from the WVS were excluded due to missing values on the items used in our analysis. After removing the missing values, the responses of 6,766 individuals were recorded. The demographic characteristics of the respondents were gathered using WVS items. These included country (V2), sex (V240), age (V242), and educational level (V248). The responses obtained from the WVS were collected from Brazil (*N* = 749), Ecuador (*N* = 1,089), India (*N* = 593), Libya (*N* = 731), Netherlands (*N* = 520), Pakistan (*N* = 784), South Africa (*N* = 1,853), and Thailand (*N* = 447); see Supplementary Table 1. The sample included 3,519 males and 3,247 females. The average age of the respondents was 39.29 (standard deviation (*SD*) = 14.65). Over 54% (*N* = 3,690) of the participants completed secondary school, over 39% (*N* = 2,651) of the participants completed a university-preparatory type of education, and over 20% (*N* = 1,364) of the participants either received some university-level education or completed university-level education with a degree.

#### Measurement

##### LH traits

We sourced and recoded WVS items conceptually similar to the Arizona Life History Battery (ALHB; Figueredo et al., 2007; Gladden et al., 2008). The ALHB indicators measure individual differences along various complementary facets of a coherent and coordinated LH strategy and converge upon a single multivariate latent construct, the K-Factor, to indicate a slow (high-K) LH strategy on the “fast-slow” continuum (Figueredo et al., 2007). The included domains from ALHB were (a) *family social contact and support;* (b) *altruism*; (c) *insight, planning and control*; (d) *religiosity*; (e) *Friend/Social support*; see Table 2 for detailed corresponding WVS questions. Higher values indicated a greater inclination for slow LH traits. The calculated Cronbach’s alpha was 0.67.

**Table 2:** The WVS items adapted from component scales of the ALHB.

Component scales of the ALHB	Corresponding WVS items
Family social contact and support	1. “V49: One of my main goals in life has been to make my parents proud” (1 = strongly agree; 4 = strongly disagree; reverse coded); 2. “V79: Tradition is important to this person; to follow the customs handed down by one’s religion or family” (1 = very much like me; 6 = not at all like me); 3. “V250: Do you live with your parents?” (1 = yes; 2 = no; reverse coded).
Altruism	1. “V74: It is important to this person to do something for the good of society” (1 = very much like me; 6 = not at all like me); 2. “V74B: It is important for this people to help the people nearby; to care for their well-being” (1 = very much like me; 6 = not at all like me); 3. “V160B: I see myself as someone who is generally trusting” (1 = disagree strongly; 5 = agree strongly; reverse coded).
Insight, planning and control	1. “V8: How important is work in your life” (1 = very important; 4 = not at all important); 2. “V75: Being very successful is important to this person; to have people recognize one’s achievements” (1 = very much like me; 6 = not at all like me); 3. “V160C: I see myself as someone who tends to be lazy” (1 = disagree strongly; 5 = agree strongly).
Religiosity	1. “V9: How importance of religion in your life?” (1 = very important; 4 = not at all important); 2. V79: “Tradition is important to this person; to follow the customs handed down by one’s religion or family” (1 = very much like me; 6 = not at all like me); 3. V145: “Apart from weddings and funerals, about how often do you attend religious services these days?” (1 = more than once a week; 7 = never, practically never); 4. V153: “Whenever science and religion conflict, religion is always right” (1 = strongly agree; 4 = strongly disagree); 5. V154: “The only acceptable religion is my religion” (1 = strongly agree; 4 = strongly disagree).
Friend/Social support	1. V5: “How important is your friend” (1 = very important; 4 = not at all important); 2. V103: “How much you trust people from people you know personally?” (1 = Trust completely; 4 = Do not trust at all); 3. “V74B: It is important for this people to help the people nearby; to care for their well-being” (1 = very much like me; 6 = not at all like me).

##### Adverse environment

We searched for items in the WVS that were conceptually related to current environmental harshness and unpredictability, which constitute the rates at which extrinsic factors cause disability and death at each age in a population (Ellis et al., 2009) and fluctuations in environmental conditions that are related to social and environmental instability (Sung et al., 2016). A general question measured perceptions of the current environment: “*In the last 12 months, how often have you or your family:*” The four responding items were starvation (V188: “*Gone without enough food to eat*”), no cash (V191: “*Gone without a cash income*”), unsafe home environment (V189: “*Felt unsafe from crime in your home*”), and no medication (V190: “*Gone without medicine or medical treatment that you needed*”). The items are rated on a four-point scale ranging from 1 (*very much*) to 4 (*not at all*). Higher values indicated a less stable current environment. The calculated Cronbach’s alpha was 0.8.

##### Justification of life-ending decisions

We measured attitudes toward ending life by examining the two WVS variables capturing beliefs about justifiable social actions, including suicide (V207) and euthanasia (V207A). Three questions assessed whether the actions of suicide and euthanasia can be justifiable and can take values from 1 (*never justifiable*) to 10 (*justifiable*), respectively, “*Please tell us for each of the following actions whether you think it can always be justified, never be justified, or something in between*.” Higher values indicated higher subjective justification scores for life-ending decisions. The calculated Cronbach’s alpha was 0.78.

#### Statistical analyses

The analyses were based on secondary data obtained from a previously published WVS dataset. First, we conducted confirmatory factor analysis (CFA) to verify the factor structure of the observed variables obtained from the WVS. Second, a correlation matrix was created to examine the relationships among variables used in the structural model. Third, The EM algorithm was selected for handling the missing data and nonresponses because it is an efficient iterative procedure to compute the maximum likelihood (ML) estimate in the presence of missing values (McLachlan and Krishnan, 2008). Finally, SEM was applied to test the structural relationships among adverse environmental conditions (harshness and unpredictability), slow LH traits (family social contact and support, altruism, insight, planning and control, religiosity, friend/social support), and subjective justification toward ending life (suicide and euthanasia). Following the recommendations of Hu and Bentler (1999), the chi-square statistic and chi-square degrees of freedom ratio (*χ2/df*) may be susceptible to overestimation of model misfit when the sample size increases. As the sample size was relatively large (*N* = 6,766) and some items were skewed, the goodness of fit index including *RMSEA*, *CFI*, and *TLI* were mainly used to examine the overall model fit.

### Results

#### Summary of CFA findings

A summary of the measurement model findings based on the CFAs of the WVS subscales is presented in Table 3. Model fit was assessed by comparing fit indices such as the CFI, RMSEA, and SRMR. However, as the chi-square statistic is known to be particularly sensitive to sample size, and given the large sample size in our study, the model may be statistically rejected despite an otherwise acceptable fit (Schermelleh-Engel et al., 2003). Model fit was classified as “good,” “marginal,” or “poor” based on these indices. The current environmental status and slow LH traits extracted from the WVS were categorized as exhibiting a “good” fit (Kline, 1998).

**Table 3:** CFA results summary for the WVS subscales in study 2.

Subscale	Cronbach’s α	χ^2^	df	CFI	RMSEA	SRMR
Current environmental status	0.8	1403.753^***^	2	0.988	0.09	0.02
Slow LH traits	0.64	940.646^***^	2	0.954	0.11	0.04

^*^p < 0.05, ^**^p < 0.01, ^***^p < 0.001. CFI, comparative fit index; RMSEA, root mean-square error of approximation; SRMR, standardized root mean square.

#### Correlation matrix and descriptive statistics

Table 4 presents the means, *SD*s, and correlations of the variables used in the SEM. The correlations were small to moderate, based on a large sample of survey data. Most current environmental conditions and slow LH trait variables were negatively correlated. Slow LH traits, except “insight,” were negatively associated with all two forms of justification for life-ending decisions. A marginal to small significant association was found between current environmental conditions and the justification of life-endingdecisions.

**Table 4:** Means, SDs, and correlations of the variables in WVS study.

	Starvation	No medicine	No cash	Unsafe home environment	Family	Friend	Altruism	Insight	Religiosity	Justify Suicide	Justify Euthanasia
Starvation	–										
No medicine	0.59^***^	–									
No cash	0.58^***^	0.60^***^	–								
Unsafe homeenvironment	0.45^***^	0.47^***^	0.36^***^	–							
Family	−0.13^***^	−0.09^***^	−0.14^**^	−0.16^***^	–	–					
Friend	−0.07^**^	−0.07^**^	−0.06^*^	−0.03^*^	0.32^***^						
Altruism	−0.06^***^	−0.03^*^	0.03^*^	−0.04^**^	0.05^***^	0.60^***^	–				
Insight	−0.16^***^	−0.15^***^	−0.09^***^	−0.10^***^	0.14^***^	0.34^***^	0.83^***^	–			
Religiosity	−0.10^***^	−0.13^***^	−0.14^***^	−0.11^***^	0.50^***^	0.22^***^	−0.04^**^	0.07^***^	–		
Justify Suicide	0.02^†^	−0.02^†^	0.01	−0.05^***^	−0.17^***^	−0.14^***^	−0.10^***^	−0.12^***^	−0.20^***^	–	
Justify Euthanasia	0.02^†^	0.03^**^	0.02^†^	0.04^**^	−0.20^***^	−0.10^***^	0.07^***^	−0.03^*^	−0.34^***^	0.57^***^	–
Mean	3.45	3.35	3.09	3.41	3.14	3.18	6.96	4.03	2.61	3.23	3.34
SD	0.86	0.92	1.03	0.89	0.71	0.53	2.28	1.26	1.08	2.76	2.97

^*^p < 0.05, ^**^p < 0.01, ^***^p < 0.001, ^†^*p* < 0.10.

#### Measurement model

**Figure 3:**
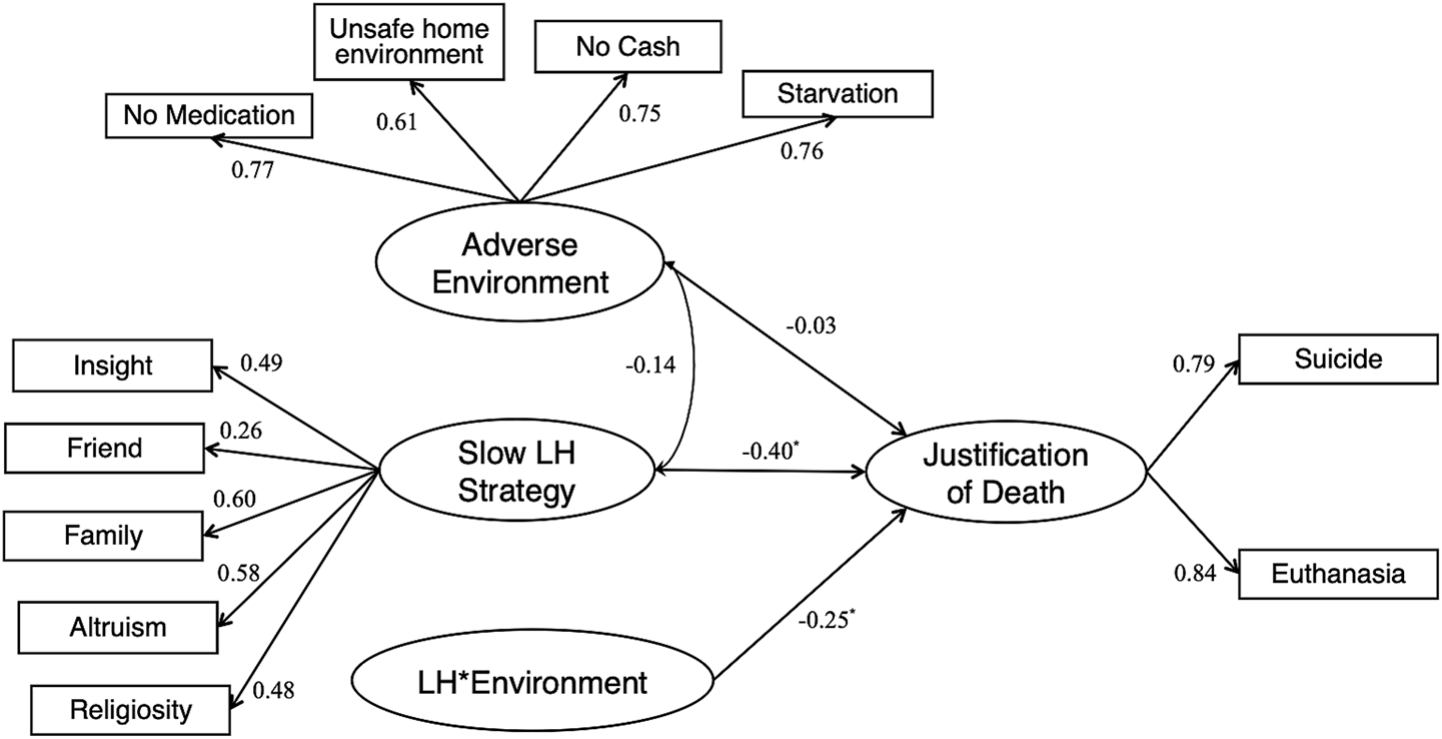
Structural relationships among slow LH traits, current adverse environment and justification of life-ending decisions. ^*^p < 0.05, ^**^p < 0.01, ^***^p < 0.001. (*CFI* = 0.984, *TLI* = 0.972, *RMSEA* = 0.041, *SRMR* = 0.022). CFI, comparative fit index; RMSEA, root mean square error of approximation; SRMR, standardized root mean square residual; TLI, Tucker–Lewis index. Higher LH values indicate a greater inclination toward slow LH; Higher values for adverse environment indicate increased levels of environmental harshness and unpredictability; Higher justification scores reflect greater subjective justification for life-ending decisions.

**Figure 4:**
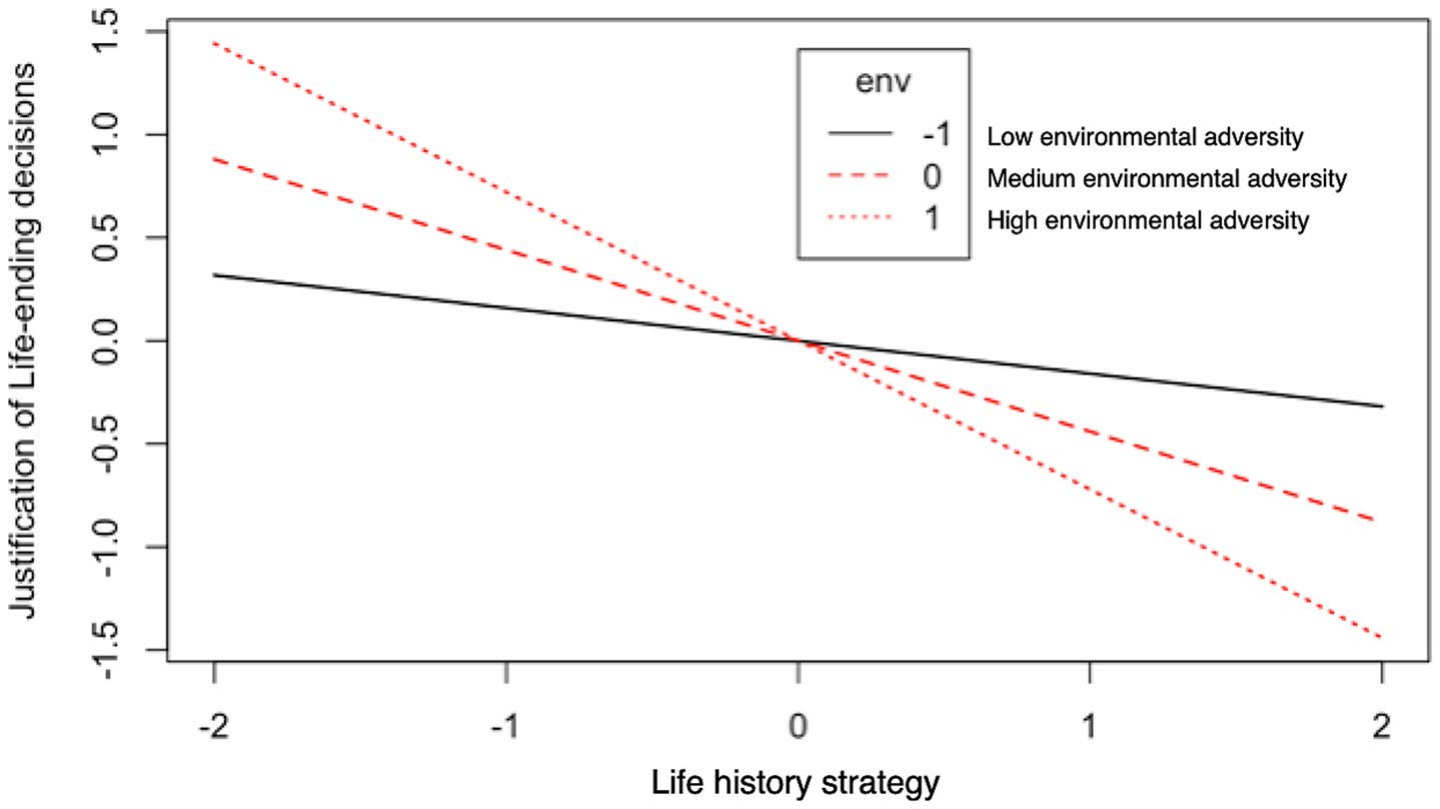
Illustration of the simple-slope analyses for the predicted justification scores of life-ending decisions resulting from the LH-by-current adverse environment interaction (Study 2). Higher LH values indicate a greater inclination toward slow LH; Higher values for adverse environment indicate increased levels of environmental harshness and unpredictability; Higher justification scores reflect greater subjective justification for life-ending decisions.

Hypothesized structural models were developed to examine the structural relationships between current environmental conditions, slow LH strategy, and the justification of life-ending decisions. The relationships between the latent constructs and indicators are shown in Figure 3. The SEM results showed that the LH strategy in a slower direction had a direct negative and significant impact on subjective justification (*β* = −0.40, *p* = 0.018), indicating that individuals who adopted a slower LH strategy believed that life-ending decisions were less justifiable. Furthermore, a negative and significant moderation effect of the current environmental adversity on the relationship between LH strategy and the subjective justification of life-ending decisions was observed (*β* = −0.25, *p* = 0.036). As shown in Figure 4, current environmental status moderated the association between slow LH strategy and the subjective justification of life-ending behaviors. Specifically, slow LH strategy predicted a less supportive attitude toward life-ending behaviors in stable environments compared to environments characterized by higher levels of harshness and unpredictability. The goodness-of-fit indices also demonstrated satisfactory results (*CFI* = 0.984, *TLI* = 0.972, *RMSEA* = 0.041, *SRMR* = 0.022).

#### Discussion of study 2

The WVS data account for individual demographic variables across multiple countries. A negative association was found between variations in LH strategy—specifically, a slower LH strategy—and the subjective justification of life-ending decisions; this effect was moderated by the current adverse environment. As postulated in previous studies, individuals who develop slow LH niches tend to be more future-oriented and focus more on collective decisions later in life. Chisholm (1993) suggested that LH strategy development was guided by an individual’s time preference, including “intertemporal choice between alternatives with varying costs or benefits over time, patience, impulsiveness, self-control, and the ability to defer gratification.” Hence, individuals’ cognitive judgment regarding life-ending decisions may be influenced by long-term versus short-term-oriented LH calibration processes. For example, slow LH strategists in predictable environments are expected to prefer long-term planning, thus showing less supportive attitudes toward life-ending behaviors because such behaviors directly jeopardize their future life and long-term outcomes. Conversely, the opposite is expected for fast LH strategists. Furthermore, not only single LH traits but also correlations between LH traits can be plastic, and different environments can change the slope or sign of the LH trait correlation (Stearns, 1992). Particularly, the interaction results indicate that harsher and more unpredictable environments beyond an individual’s control may alter attitudes toward life-ending behaviors, demonstrating the interplay between LH strategy and current environmental conditions. Despite various indicators of LH strategy converging on a single multivariate construct, the latent K-factor (e.g., ALHB and mini-K), the scale developed from WVS may be accompanied by decreased precision in measuring LH traits. Therefore, more research will be needed to support theoretical prediction and, more importantly, the precise and direct measurement of LH strategy.

## Conclusion

The detailed analysis of life-ending scenarios in Study 1, along with the replication using the larger WVS dataset in Study 2, highlights the variation in life-ending decisions as predicted by LH theory. This study extends previous research by broadening the evolutionary perspective and applying LH theory to life-ending decisions, specifically examining how an individual’s LH strategy and current environment influence attitudes toward life-ending behaviors. People differ in their awareness and justification of dying and life-ending actions, and, unlike other major life events such as birth, the psychological mechanisms underlying death and dying from an evolutionary perspective are rarely explored. The considerable variation in subjective justification for suicide and assisted suicide (euthanasia) suggests that decisions related to immediate adaptive problems are shaped by LH tradeoffs and environmental conditions.

This study proposes and analyzes a moderation-SEM to examine how the interaction between LH strategy and current environmental adversity influences the subjective justification of life-ending behaviors. This assumption is based on the idea that individuals’ justification of life-ending decisions may arise from LH calibration processes within the context of moral decision-making (Zhu et al., 2019). To adapt to environmental changes and maximize fitness, individuals develop LH strategies aimed at optimal adaptation to their current environment; however, empirical research on the flexibility and adaptive functionality of this phenotypic plasticity remains limited (Nettle and Frankenhuis, 2019). The present research integrates LH theory in an exploratory analysis of life-ending decisions, demonstrating that individuals who adopt a slower LH strategy exhibit less justification for suicide and euthanasia (Study 2), and that current environmental adversity moderates this effect (Studies 1 and 2). Both studies confirm that cognitive judgments regarding suicide and euthanasia are influenced by the interaction between individuals’ previously calibrated LH strategy and their current environmental conditions. Notably, Study 1 and Study 2 revealed different patterns in the moderation effect. In Study 1, individuals with a slow LH strategy were found to view life-ending behaviors as less justified than those with a fast LH strategy at high levels of environmental adversity; however, this association was reversed at low levels of environmental adversity. In the population-based Study 2, we observed a difference in the strength of the interaction effect. At high levels of environmental adversity, individuals with a slow LH strategy were less likely to justify life-ending behaviors compared to those with a fast LH strategy; this difference diminished at lower levels of environmental adversity. Based on the evolutionary mismatch hypothesis, psychological adaptations are mechanisms that receive environmental cues as input, process this information using evolved decision-making rules, and generate adaptive thoughts, attitudes, and behaviors as output (Li et al., 2017). Our findings support the idea that processing information about mortality leads to flexible psychological and behavioral adjustments, which in turn influence LH strategies and their expressions. Given the complexity of the moderation effect and the interplay between these factors and their sublevels, our results highlight the need for clearer guidelines on interpreting interaction effects in future research.

Applying the LH framework to study the psychology of death and life-ending matters allows the shift in focus within LH theory in psychology to LH strategic responses to individual environmental variables (Nettle and Frankenhuis, 2020). Previous research has established a framework for developing interventions that focus on LH traits, particularly in domains such as investment in different life components, specifically, forgoing versus delaying (Lu and Chang, 2019), as well as behaviors and psychological dispositions that facilitate adaptive responses to various ecological conditions (Guo and Lu, 2024; Simpson et al., 2012). These findings align with earlier studies linking environmental threats to LH strategies (Chisholm, 1993; Greenberg et al., 1992; Rosenblatt et al., 1989). The present study suggests that the moderating effect of current environmental adversity on LH strategies may reflect the flexible adjustment of behavior in response to short-term changes in local conditions (Griskevicius et al., 2011a; Pepper and Nettle, 2017). Simple-slope analysis of the moderation effect demonstrates that environmental harshness and unpredictability directly influence LH traits through their interaction effects on the justification of life-ending decisions, either by shaping LH strategies or by regulating individuals’ cognitive judgments on these issues. This interaction indicates that current adverse environments further reinforce the association between individuals’ previously calibrated LH strategies and their attitudes toward life-ending behaviors, thereby strengthening cognitive decision-making related to these behaviors. Therefore, further research is needed to explore additional underlying mechanisms that may explain both the direct and indirect impacts of LH tradeoffs on the psychology of death.

We found a negative association between slow LH orientation and the subjective justification of life-ending decisions in Study 2. This result aligns with previous research showing that slow LH strategies are associated with pursuing long-term outcomes (Nettle, 2010) and greater cognitive and behavioral control (Gladden et al., 2009). One possible explanation for this finding is that individuals with a slow LH orientation prioritize future outcomes, such as long-term thriving and survival. Therefore, making immediate life-and-death decisions or ending a life is less favorable for these long-term-oriented individuals. In contrast, those with a faster LH strategy tend to be less future-oriented and more pessimistic about their future (Copping et al., 2013). As a result, an intuitive cognitive style and reliance on heuristics may lead them to avoid time-intensive reflection and disregard future outcomes in threatening situations (Wang et al., 2022). Although humans have a survival instinct, there is variability in individual LH tradeoffs, which is reflected in cognitive styles as well as behavioral and psychological manifestations. Using a large and representative sample in Study 2, we identified correlations between LH manifestations and life-ending judgments. Slow LH strategies are typically adopted in stable and predictable environments that signal greater resource investment and a higher likelihood of future fitness payoffs (Belsky et al., 2012). When faced with mortality threats and life-ending dilemmas, individuals with a slow LH orientation are likely to be more optimistic about future outcomes than those with a fast LH orientation, and consequently, they exhibit less justification for suicidal attempts and euthanasia practices.

We extend this body of work on cognitive judgment by examining life-ending decisions, which are, by definition, referred to as the process in which a decision is made after reflection on the consequences of that choice (Kahneman, 2003). This assumption also reflects the long-term (slow) versus short-term (fast) LH tradeoffs. Recent work highlights that variations in LH strategy may play an essential role in shaping cognitive decision-making by considering adaptive tradeoffs, such as forgoing versus delaying gratification and short-term versus long-term orientation, as well as the functions and costs of heuristic versus systematic cognitive styles (e.g., Wang et al., 2022). Overall, the results from Studies 1 and 2 are compatible with a wide range of evolutionary mechanisms and may have practical implications for educational programs, including mitigating death-related fear and anxiety, coping with death-related issues, and suicide prevention. A stable early environment is crucial for shaping LH strategy in adulthood and fosters a long-term orientation when making decisions in emergency situations. Family counseling and social services can provide protective barriers against early life adversity and help build healthy family functioning and social environments. Our findings also suggest that situational adaptation can benefit from some degree of environmental adversity and uncertainty. This implication provides insights for future intervention programs by considering the importance of environmental adaptation.

This paper had several limitations. First, the cross-sectional design of Study 1 and Study 2 may have limited the ability to test moderating effects, restricting a detailed exploration of possible factors in specific situational contexts. Future research should employ longitudinal designs to examine the roles of other potential mediating and moderating factors, such as family structure, socioeconomic status, culture, religion, and local environment. Second, the life-ending scenarios used in Study 1 require further examination for conceptual validity. Both suicidal behavior and assisted suicide refer to life-ending actions, but they also encompass a range of suicide attempts—from high-lethality to low-lethality (Sher and Oquendo, 2023)—as well as various cognitive styles (Beautrais et al., 1999). The accuracy and level of detail in the vignettes depend on the descriptions of life-ending scenarios, which cannot capture all possible aspects of the hypothetical experience. More standardized vignette-based methods are encouraged in future studies. Additionally, the acceptability of life-ending actions and access to euthanasia vary across cultures and religions. Therefore, future research should explore problem-solving and cognitive styles among individuals exhibiting suicidal behavior in greater depth to better understand their thought processes and develop targeted interventions. Third, this study focuses on how individuals estimate the current environmental state in a general sense, specifically their exposure to environmental unpredictability and harshness during adulthood. Given this narrow scope, the measurement of environmental unpredictability and harshness depends on whether the environment is stationary or non-stationary, which may reduce the precision of estimating perceived extrinsic mortality risk. Current research trends highlight the need for a dimensional approach to environmental factors that shape individuals’ LH strategies (Ellis et al., 2022). Future analyses should examine a broader range of environmental contexts across multiple dimensions of environmental experience. When operationalizing environmental harshness, it is also recommended to consider additional sources of morbidity and mortality (Baldini, 2015; Yang et al., 2022). Fourth, self-reporting questions may underestimate or overestimate the causal relationships between LH strategy, the current adverse environment, and subjective justification scores on life-ending decisions. In current scenarios, it is impossible to include all possible variables and their nuances, such as hypothetical medical treatments, legal issues, social environment, SES, and cultural factors. Hence, future studies could explore the ways of integrating evidence across different cultures and local social environments. The estimates of a population value vary considerably across samples, becoming increasingly less precise as sample size decreases. The small sample size in Study 1 (*N* = 147) may limit the ability to detect small effect sizes and can lead to less precise estimation of effect sizes. Moreover, the constructs or survey instruments utilized in the WVS may not possess equivalent meanings or psychometric properties across diverse cultural settings (Desa et al., 2018). Therefore, future cross-cultural research should prioritize the adaptation or development of culturally sensitive measurement tools, incorporating qualitative pre-testing to ensure that constructs are understood and interpreted consistently across different contexts. Fifth, though the current standard measurement of LH strategy, the latent K-factor (e.g., ALHB and mini-K), is employed in many published studies, concerns are raised for the K-factor as the clusters of certain psychosocial traits into meaningful functional composites of LH strategy (Gruijters and Fleuren, 2017). According to the critiques raised by Nettle and Frankenhuis (2020) and Sear (2020), the predictions throughout development are varying and intercorrelated in the broader suits of LH traits (e.g., behavioral, motivational, and attitudinal traits) and psychological manifestations (e.g., personalities). Future research should incorporate more sophisticated measures of LH-related traits. Finally, Study 2 selected WVS items that were conceptually similar to previous scales and an *a priori*-defined criterion. To improve the validity of future scales, future studies should include multiple informants and a broader range of survey items and explore the wider physical and mental health consequences of life-ending decisions to inform the development of effective interventions.

There is ongoing debate about whether suicide or deliberate self-killing is an evolutionary by-product with adaptive value (e.g., Preti, 2011; Soper, 2018). The inherent risk of maladaptation may arise if individuals’ perceptions or forecasts about their environment are inaccurate (Kavanagh and Kahl, 2018). Previous research has primarily relied on self-referential predictions without critically examining the assumption of adaptive responses (Zietsch and Sidari, 2020). Therefore, further investigation into the demographic and psychosocial correlates of life-ending actions, as well as the stochastic influences and variability of suicide risk, is needed. Such knowledge would enhance interventions targeting environmental and social factors. It is also essential to recognize differences in attitudes and the adaptive functions of LH strategy to reduce potential life-ending actions. Despite the aforementioned limitations, this study offers several new approaches to examining the psychological mechanisms underlying suicide and physician-assisted suicide (euthanasia), and identifies a significant moderating effect of current adverse environments (Studies 1 and 2). The results suggest that a slow LH strategy is associated with less subjective justification and acceptability of life-ending decisions (Study 2), whereas a fast LH strategy is linked to greater subjective justification and acceptability. Furthermore, exposure to current environmental adversity strengthened the association between LH strategy and subjective justification of life-ending decisions.

## Data Availability

The raw data supporting the conclusions of this article will be made available by the authors, without undue reservation.

## Footnotes

^1^ https://www.r-project.org/
