# Supplementary material for: Life-history strategy, adverse environment, and justification of life-ending decisions
Source: Front Psychol. 2025 Jul 21;16:1568204. doi: 10.3389/fpsyg.2025.1568204 (PMC12318959; doi:10.3389/fpsyg.2025.1568204)
Supplement: Supplementary file 1 [file Table_1.docx]

**Supplementary Table 1**

*List of countries and the average justification scores of life-ending behaviors in Study 2*

| Country | Total participants | Average justification score of suicide | Average justification score of euthanasia |
| --- | --- | --- | --- |
| Brazil | 749 | 1.60 | 2.47 |
| Ecuador | 1,089 | 1.90 | 2.41 |
| India | 593 | 3.90 | 3.84 |
| Libya | 731 | 1.51 | 1.73 |
| Netherlands | 520 | 4.82 | 7.64 |
| Pakistan | 784 | 1.52 | 1.63 |
| South Africa | 1,853 | 4.47 | 4.74 |
| Thailand | 447 | 1.61 | 4.09 |

*Note*. Higher justification scores indicate higher subjective justification scores for life-ending decisions.
