# Supplementary material for: Life-history strategy, adverse environment, and justification of life-ending decisions
Source: Front Psychol. 2025 Jul 21;16:1568204. doi: 10.3389/fpsyg.2025.1568204 (PMC12318959; doi:10.3389/fpsyg.2025.1568204)
Supplement: Supplementary file 2 [file Table_2.docx]

**Appendix A**

Vignettes used in Study 1^[[1]](#footnote-1)^

**1. Suicide vignettes**

*Scenario 1*. You are divorced, living alone, and admitted to the hospital in a nearly comatose condition yesterday because of an overdose of approximately thirty tablets of Valium, five MGM, combined with alcoholic intoxication. A heavy drinker, you have been unemployed from your janitorial job for the past three months because of your drinking. You have been feeling increasingly depressed since being fired, and for the past two weeks, you had insomnia, anorexia, and weight loss. The use of alcohol has increased considerably in the past month. You don’t have any close relationships or care how others would feel.

After another lay-off and you are running out of money and can’t afford to rent an apartment, you choose to: ^[[2]](#footnote-2)^

A. You will continue to live and not choose suicide (suicide probability 0%).

B. You are kind of considering suicide (suicide probability 20%).

C. You are seriously considering suicide (suicide probability 40%).

D. You are likely to commit suicide (suicide probability 60%).

E. You are very likely to commit suicide (suicide probability 80%).

F. You decide to commit suicide (suicide probability 100%).

Do you think this suicidal behavior is justified?^[[3]](#footnote-3)^

A. Very justifiable

B. Justifiable

C. Neutral

D. Unjustfiable

E. Very unjustifiable

*Scenario 2*. You feel trapped and can’t cope at home with your schizophrenic symptoms. You want to be in the hospital and continue to feel like killing yourself. Your spouse indicates that you have threatened to shoot family members - recent arrest for disorderly conduct (threatened police with a butcher knife).

Your symptoms could not be alleviated, you choose to:

*Scenario 3*. You constantly fight with your partner, leading to a recent breakup (there is a long history of mutual verbal/physical abuse). You cannot afford any separation because you do not have a job, your partner has been manipulating you for a long time, and your partner believes you will come back because you don’t have any income. You always feel tearful, shaking, frightened, and hopeless, seldom leave home, and are at high risk for physical violence that may cause permanent injury.

Your life is ruined by this relationship, you choose to:

*Scenario 4*. Two years ago you began using pot and drinking. At first you confined these activities to the weekends. Gradually you began to use harder drugs. You could not start the day without drugs. You were experiencing difficulty in learning, had lost weight because of lack of appetite and really suffered from side effects from drugs.

After few days of insomnia and mental breakdown, you choose to:

*Scenario 5*. You were in an automobile accident last year. You had been a member of the university’s freshman basketball team and were very active in school activities. The accident left you permanently paralyzed from the waist down.

After you realize your conditions, you choose to:

*Scenario 6*. You were a senior in college with a student loan. You had wanted to be a layer for four years. Your grades had been failing consistently each semester. Last week you got back your LSAT scores and found that they were so low that you probably would not have been admitted to any law school.

After you find out that you might not graduate with a bachelor’s degree, you choose to:

*Scenario 7*. Your partner died last year. You had felt depressed and lost since your partner’s death. When you thought of your partner, you were overwhelmed by sadness and hopelessness.

You are convinced that you would never be happy again, you would choose to:

*Scenario 8*. For many school years you had been teased by your friends and classmates because of your body size. Recently, your face had broken out with severe acne, causing fellow students to bully you and tease you about your skin as well as your weight.

After a fight with your classmates, you choose to:

*Scenario 9*. You were driving your family’s car to a college basketball game. The night was rainy. You didn’t see a truck stalled across the road until you were almost on it. In the resulting accident, one of your passengers was skilled and another was crippled.

You can’t get over your feelings of guilt and sorrow about the accident, you choose to:

**2. Euthanasia vignettes**

*Scenario 1*. You were cycling down a hill when you collided with an oncoming bicycle around a blind curve, catapulting you onto the mountain path. Your helmet cracked just above the left temple, and fell directly on your head, and your body followed in a grotesque somersault that broke your neck at the top of the spine. You suffered a grievous injury leading to a terminal or vegetative state. You wanted no procedures done that “would serve only to prolong the moment of my death unnaturally and to postpone or prolong the dying process unnaturally.”

If you get a chance to choose before surgery, you choose to:^[[4]](#footnote-4)^

A. You will continue to live and not choose euthanasia (euthanasia probability 0%).

B. You are kind of considering euthanasia (euthanasia probability 20%).

C. You are seriously considering euthanasia (euthanasia probability 40%).

D. You are likely to proceed euthanasia (euthanasia probability 60%).

E. You are very likely to proceed euthanasia (euthanasia probability 80%).

F. You decide to proceed euthanasia (euthanasia probability 100%).

Do you think euthanasia is justified?^[[5]](#footnote-5)^

A. Very justifiable

B. Justifiable

C. Neutral

D. Unjustifiable

E. Very unjustifiable

*Scenario 2*. You have recently been diagnosed with advanced colon cancer. You have been hospitalized with pneumonia that developed after your last doses of chemotherapy. Your doctor told you about the seriousness of your illness and sadly explained the fact that you may have little time to live.

If you get a chance to choose before your next painful chemotherapy, you choose to:

*Scenario 3*. You were profoundly overweight and drank excessively every night. Earlier in your life, you had experimented with illicit injectable drugs. Recently, you had been fatigued and noticed that your abdomen constantly looked distended. You also saw that you would bleed profusely, even with minor cuts. When you finally went to the doctor, the doctor told you that you were in an advanced stage of Hepatitis C and your condition was terminal. You couldn’t change your lifestyle because of your addiction to alcohol and drugs, and your state is getting worse, and you feel like you may only have little time in life.

If you get a chance to choose before you hospitalized, you choose to:

*Scenario 4*. You had had leukemia since you were 18. You were now 20. You had known for over a year that the disease was most likely going to kill you. Lately the pain have increased and the drugs no longer controlled it, you choose to:

*Scenario 5*. You had breast cancer with metastases. Despite undergoing several treatments, your disease is no longer curable. You were in severe pain that cannot be sufficiently relieved. In addition, you disliked the feeling of loss of control that you experienced. In your working days, you always felt in control. You cannot take it anymore, you choose to:

*Scenario 6*. You have been suffering from severe depression for years, and your psychiatrist’s treatment has not worked. You regularly tell your physicians that you want to die. You already have had one unsuccessful suicide attempt. You decide to re-visit your psychiatrist, you choose to:

*Scenario 7*. You suffer from early dementia and sometimes you are forgetful. You fear what is to come, the progressive loss of memory and the moment you will not recognize your surroundings anymore. Your own mother suffered from severe dementia, and you absolutely do not want to experience this process yourself. You decide to re-visit your general practitioner, you choose to:

*Scenario 8*. You used to be a professor at the university. You enjoyed your life at that time. You neither married nor had children. Now you have grown old; many of your friends died. You often feel lonely. You are in good physical and mental condition. Though you are aware that you could live for many years, you fear this loneliness, you choose to:

*Scenario 9*. You are 62 years old and suffering from dementia. You don’t recognize your wife and children anymore, refuses to eat and withdrawals into yourself more and more. It is no longer possible to communicate with you about your treatment. Shortly before you became demented, you choose to:

1. All vignettes ware translated in local language. [↑](#footnote-ref-1)
2. This question will be asked after reading the scenario. [↑](#footnote-ref-2)
3. This question will be asked after reading the scenario. [↑](#footnote-ref-3)
4. This question will be asked after reading the scenario. [↑](#footnote-ref-4)
5. This question will be asked after reading the scenario. [↑](#footnote-ref-5)
